# Accidental Contamination with Oil during Endodontic Surgery

**DOI:** 10.22037/iej.2016.18

**Published:** 2016

**Authors:** Hugo Plascencia, Mariana Díaz, Patricia Cholico, Monserrat del Real, Salvador Márquez-de Alba

**Affiliations:** a * Endodontic Postgraduate Program, CUCS-CUAltos, University of Guadalajara, Mexico; *; b * Endodontic Postgraduate Program, CUCS, University of Guadalajara, Mexico; *; c *Endodontic Postgraduate Program, CUAltos, University of Guadalajara, Mexico*

**Keywords:** Endodontic Surgery, Histopathological, Tissue Contamination

## Abstract

The modern surgical endodontic treatment is a safe and predictable procedure with high success rate. However, several factors can retard or impede the proper healing process. Use of a high speed handpiece during hard tissues management (osteotomy and apical resection) can potentially be one of these factors. Formation of metallic debris from the surgical diamond burs, production of necrotic local tissue due to overheating and the direct liberation of air from conventional handpiece into the working area are potential irritants able to delay the tissue healing. The aim of the present article is to report the histopathological findings of the trans-operational accidental contamination with oil in the surgical area during an endodontic surgery.

## Introduction

The modern surgical endodontic treatment is a safe and predictable procedure, which according to the systematic review by Tsesis *et al.* [1], offers a success rate of 89% after 1 year. However, several factors can retard or impede the proper tissue healing process, such as the inappropriate use of a high speed handpiece during the hard tissues management (osteotomy and apical resection). In literature, it has been reported that the direct liberation of air to the working area [2-4], the creation of metallic debris from surgical diamond burs [5] or the formation of necrotic bone tissue as a result of overheating [6, 7], are among the potentially damaging phenomena generated by the incorrect usage of high speed engines. The objective of this article is to report the histopathological findings of the trans-operational accidental contamination with oil in the surgical area during endodontic surgery.

## Case Report

**Figure. 1:**
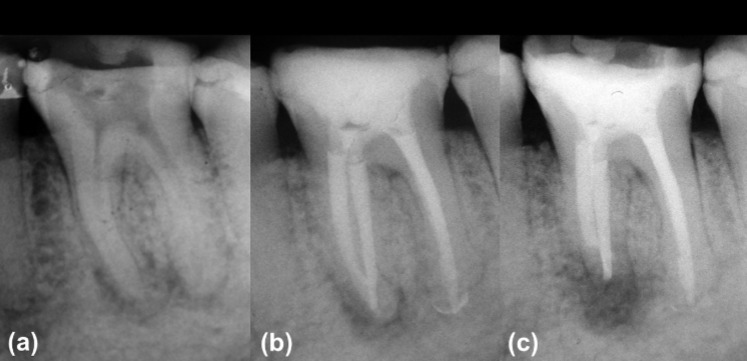
*A*) Initial radiograph, *B*) Non-surgical endodontic post-treatment radiography*, C*) Endodontic post-surgery radiography

A female 46-year-old patient was referred to the Endodontic Department of the University of Guadalajara, Mexico, for evaluation of her left mandibular first molar. The chief complaint was moderate spontaneous pain that increased upon biting. The patient was subjected to endodontic emergency treatment 1 year earlier, but she never went back to finish the procedure. The medical history revealed no history of systemic problems or allergies. Upon clinical inspection, the access cavity was found without any restoration and the pulp chamber was exposed to the oral fluids. No response was elicited upon buccal or lingual mucosa palpation, contrary to the intense pain caused by horizontal and vertical percussion. The probing depth and mobility were within the normal limits. The radiographic examination revealed narrow and curved root canals, which were surrounded by a diffuse periapical lesion of approximately 6 mm in width and 4 mm in length on the apical portion of the mesial root (Figure 1A). Therefore, once the clinical and radiological findings were collected, a pulpal diagnosis was established of previously initiated endodontic treatment with signs of infection and a periapical diagnosis of acute alveolar abscess. Considering the uncertain initial prognosis due to suspected persistent endodontic infection and pulp chamber exposure to saliva, a two-visit endodontic root canal retreatment was scheduled. 

Once signing a written informed consent by the patient, regional anesthesia was applied by buccal infiltration with Articaine (Articaine with 1:100000 epinephrine; Septodont, Saint-Maur-des-Fossés, Cedex, France). The left mandibular first molar was isolated with rubber dam and the decayed tissue was completely removed prior to access cavity preparation. The whole treatment procedure was performed using dental operating microscope. The orifices of three root canals (including mesio-buccal, mesio-lingual and distal canal) were identified with a DG-16 endodontic explorer. The operatory field was disinfected with gauze soaked in 5.25% sodium hypochlorite (NaOCl, Clorox, Clorox de Mexico S.A. de C.V., Tlanepantla, México) and the root canals were explored with a #15 K-file and abundant 3% NaOCl irrigation. Cervical flaring was carefully performed with #2 and #3 Gates Glidden drills (Dentsply, Maillefer, Ballaigues, Switzerland). After determining the working length with radiography, the cleaning and shaping of the apical thirds were completed with S1, S2 and F1 Universal ProTaper rotatory files (Dentsply, Maillefer, Ballaigues, Switzerland), irrigating with 3% NaOCl between the instruments. The mesial and distal canals were manually widened up to #40 and 60, respectively using hand K-Flexofiles (Dentsply Maillefer, Ballaigues, Switzerland). Calcium hydroxide paste was placed as an intracanal dressing and the coronal access cavity was temporary sealed.

After 7 days, the patient returned with persistent symptoms, especially acute pain upon percussion in the mesial zone of the treated molar. Therefore, it was decided to refresh the intracanal calcium hydroxide. Ten days later, the signs and symptoms were still present. As a result, it was decided to complete the endodontic treatment and schedule a complementary surgical procedure. When the intracanal medication was removed, a flow of purulent exudate was present in the mesial root canals. In response, they were abundantly irrigated with 5.75% NaOCl and were dried with absorbent paper points. All the canals were filled with gutta-percha embedded in a calcium hydroxide sealer, using the Tagger technique. The access cavity was sealed with temporary cement and a final radiography was taken (Figure 1B).

***Endodontic surgery***

Ten days later, inferior alveolar nerve block with two cartridges of articaine (Medicaine 1:100000, Septodont, Saint-Maur-des-Fossés, France) was performed and after waiting 15 min for anesthesia, a full-thickness triangular flap was raised. Access to apex of the mesial root was performed using a #4 long shank round carbide bur mounted on a conventional high speed handpiece, under abundant irrigation of saline using a hypodermic syringe. After locating and exposing the periapical lesion, all the tissue within the bone crypt was enucleated with a #86 Lucas curette and then was immediately submerged in 10% formalin solution for fixation and subsequent routine histopathology. Once the bleeding was under control by the use of gauze soaked in ferric sulphate, the remaining 3 mm of the root apex was cut with a Zecrya bur (Dentsply, Maillefer, Ballaigues, Switzerland) mounted on a high speed handpiece, accompanied with saline irrigation. The ultrasonic retrograde preparation was made with ultrasonic tips (Endo retrograde Kit, NSK, Brasseler, USA), to low intensity and abundant saline irrigation was provided drop by drop at distance. After drying the retrograde cavity with absorbent paper points, MTA was transported using MAP system (Produits Dentaires, Vevey, Switzerland) and compacted in both mesial canals, followed by radiographic verification (Figure 1C). The flap was repositioned and sutured with 5-0 nylon separated stitches. Finally, postoperative instructions were given orally and written. Five days later, the patient returned for suture removal and did not report any unusual discomfort. Clinical and radiographic follow-ups were not possible due the patient’s migration to another country.

***Histopathological analysis of the sample***

**Figure. 2:**
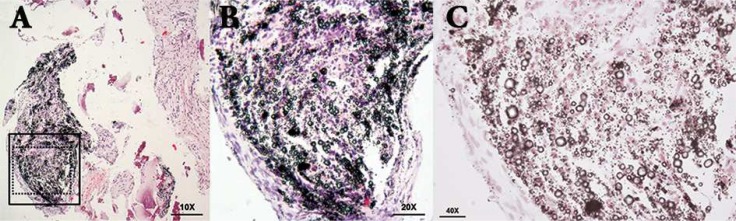
Photomicrographs showing the sections from the periapical lesion stained with Hematoxylin and Eosin; *A)* Low magnification view (10× magnification) showing two type of tissues, one contamination free *(right side)* and the other with foreign agents *(left side)*; *B)* Another view (20× magnification) of the remarked zone in the black square of previous image*,* where it can be observed some particles deposited over the sample surface; *C)* Photomicrograph of the remarked zone in the black dotted square (40× magnification), where are visible multiple birefringent deposits in vacuoles and drop shape of diverse diameters that are compatible with oil

In randomized microscopic evaluation of micro-slices of the sample (Figures 2A and 2B), granulomatous tissue hyperplasia was observed as well as chronic inflammatory infiltration of mononuclear cells, histiocytes and plasma cells were found in the stromal fibrous-connective tissue, in which collagen fibers were loose and irregularly arranged. Moreover, in the peripheral fields of the sample, there were visible multiple birefringent deposits with variable diameters and drop-shape, presumably indicative of oil splashing through the handpiece that liberated air in the working area and contaminated the area during hard tissues management. Therefore, the histopathological diagnosis was granulomatous tissue with exogenous oil deposits.

## Discussion

To the best of our knowledge, this case is the first histopathological evidence that reports the presence of oil contamination within the enucleated periapical granulomatous tissue which must be integrated to the contraindications of use of conventional handpiece in any dental surgical intervention. 

It is well demonstrated that non-surgical root canal treatment is a reliable therapy in short- and long-term. According to Salehrabi and Rotstein [8], high percentage (97%) of endodontically treated teeth remain in the oral cavity after 8 years. However, the authors reported that the remaining 3% failed before getting to the third year after the treatment. For those failed cases, the non-surgical endodontic retreatment becomes the first option, because it is a conservative intervention and offers a high percentage (89%) of tooth retention after 5 years of monitoring [9]. However, for the group of cases where none of the previously aforementioned small therapies were successful, there is the possibility of preserving these teeth with endodontic surgery, a predictable procedure with a high level of evidence for notable (>89%) success rate [1].

During an endodontic surgical procedure, some complications could occur that may retard or prevent the adequate periapical healing. Various operational accidents related with inappropriate use of the high speed handpiece have been reported. Formation of metallic debris from the surgical diamond burs [5] or production of necrotic local tissue due to overheating [6, 7], are irritants able to delay tissue healing. Nonetheless, the incidents related from the direct liberation of air from non-surgical handpiece into the working area could jeopardize the patient’s life due to formation of subcutaneous or pneumomediastinum emphysema [2-4]. 

A precautionary measure to avoid these such events is the use of a surgical high speed handpiece. This device has a 45^°^ angled head which improves operator’s visibility, although it’s principal advantage is the rear air liberation, including the oil. Furthermore, the application of ultrasonic devices and more recently piezoelectric instruments during the endodontic surgery, allow the management of bone tissue and the root apical third in a safe way. This last system is extremely advantageous because it has the ability to cut the hard tissues (bone and root) without affecting the soft tissues (blood vessels, nerves and mucosa) [10]. However, more clinical studies need to be done to confirm the superiority of this tool over traditional techniques.

## Conclusion

This case shows histopathological evidence of operational accidental contamination of periradicular tissues with oil during endodontic surgery, related with the use of high speed nonsurgical handpiece with direct air liberation towards the working area.
